# Revisiting the Mechanisms Involved in Calcium Chloride Induced Bacterial Transformation

**DOI:** 10.3389/fmicb.2017.02169

**Published:** 2017-11-07

**Authors:** Azka Asif, Hareem Mohsin, Rabia Tanvir, Yasir Rehman

**Affiliations:** ^1^Department of Microbiology and Molecular Genetics, University of the Punjab, Lahore, Pakistan; ^2^University Diagnostic Lab, Department of Microbiology, University of Veterinary and Animal Sciences, Lahore, Pakistan

**Keywords:** calcium chloride, transformation, *Escherichia coli*, DNA, competent cells

Bacterial transformation is a crucial part of cloning process and has been widely used in many studies (Swords, 2003; Gigova et al., 2006). The mechanism is marked by two phases, the first phase involves the uptake of the DNA across the cellular envelope and the second phase involves the setting up of the DNA in the cell as a stable genetic material (Hanahan, 1983). The procedure of transformation is of physicochemical nature rather than strictly being a chemical or a physical procedure, since the cells are manipulated with cations (or a combination of cations) and temperature imbalances to render them competent to uptake foreign DNA. It is a common understanding that the chemical manipulation is linked to the induction of competency while the physical manipulation is linked to the uptake of foreign DNA. The two methods act together to bring about the act of “artificial DNA internalization.”

Over time various methods have been used to make cells competent such as by using dimethyl sulfoxide (DMSO), divalent cations, or polyehtylene glycol (PEG) (Klebe et al., 1983; Chan et al., 2013). Other than these chemical methods, electroporation has also been tested and used to induce competency (Dower et al., 1988; Yoshida and Sato, 2009; Liu et al., 2013). The use of divalent cations has been the most effective chemical treatment to bring about transformation (Day, 2004). Among various cations, divalent calcium cation (Ca^2+^) has proven to be the most effective one (Weston et al., 1981) both alone (Dagert and Ehrlich, 1979) and in various combinations. A combination of divalent and monovalent ions, such as calcium and magnesium (Taketo, 1974; Wensink et al., 1974), calcium and manganese (Enea et al., 1975), calcium, rubidium, and dimethyl sulfoxide (Kushner, 1978) and other alkali metals with a prolonged incubation at 0°C (Taketo, 1972; Dagert and Ehrlich, 1979) has also been reported to be effective (Roychoudhury et al., 2009). Generally, all divalent cations enhance the transformation process. Hanahan (1983) found that the presence of magnesium in bacterial culture media increases the transformation efficiency by 15- to 20-folds as compared to the cells grown in the absence of magnesium. He also observed that the addition of magnesium in the media 30 min before the time of collection of cells also enhances the transformation up to ~60%. However, addition of magnesium as the cells are harvested and incubated on ice enhances transformation up to ~40%. The addition of calcium or manganese ions has also shown almost the same stimulatory effect as that of the magnesium ions (Hanahan, 1983). However, incubation time with calcium chloride or any other divalent cation for that matter, should be optimized. It was observed that a period of 24 h incubation in cold calcium chloride makes the bacterial cells 20–30 times more competent and 4–6 times more efficient for transformation as compared to the cells that are obtained immediately after CaCl_2_ treatment (Blattner et al., 1977; Dagert and Ehrlich, 1979). Curtiss et al. (1977) experimented on *E. coli* strain X1776 and observed the effect of various set of conditions on the efficiency of transformation. He treated the bacterial culture with a combination of manganese, calcium, rubidium, and potassium ions along with DMSO and sucrose at 0°C followed by a heat pulse at 42°C. However, these conditions did not give successful results when other strains of *E. coli* were used (Hanahan, 1983). Meselson and Yuan (1968) found these conditions promising for successful transformation in case of a strain of *E. coli* MM294 than the standard “calcium chloride” protocol. Bolivar et al. (1977) reported 10^6^ transformants when the cells were treated with calcium alone while Kushner (1978) reported to obtain 10^7^ transformants after treating the cells with rubidium along with calcium chloride. Norgard et al. (1978) was also able to obtain 10^7^ transformants with the method followed by Kushner (1978) in case of K-12 strain X1776 of *E. coli*. However, transformants yield varied from specie to specie and strain to strain (Mercer and Loutit, 1979). It was reported by Sjöström et al. (1972) that the optimum concentration of calcium chloride for the uptake of DNA by *S. aureus* is 0.1 M CaCl_2_. The repulsion between foreign DNA and the bacterial cell, owing to negative charges on them both, are overcome by these divalent cations. This is applicable for linear DNA fragments as well as circular DNA molecules such as plasmids (Mandel and Higa, 1970; Tsen et al., 2002). It is thought that the divalent cations bind both to the cell and the DNA, thus neutralizing the charge altogether. The calcium bound to the DNA further helps the DNA to adsorb to the competent cell (Panja et al., 2008b). Moreover, DNA binding proteins present in the cell membrane could also aid in this interaction. The anchorage of the DNA to the membrane eliminates the risk of detachment or expulsion of DNA (Clark et al., 2002). Further, the low temperatures used in transformation protocols congeals the lipid moiety and consequently restricts the fluidity of the cell membrane which strengthens calcium-cell surface interaction. In this way calcium ions, bound with cell surface as well as the foreign DNA, brings the DNA to the cell. Clark et al. (2002) showed that the relative association of divalent cations (e.g., Ca^2+^) is more with the cell membrane as compared to its association with foreign DNA, whereas certain trivalent cations (e.g., spermidine) interact more readily with the DNA (Li et al., 2004). It was also reported in this study that Ca^2+^ has more pronounced role to play in development of competency as compared to spermidine or trivalent cations (Clark et al., 2002). Membranes absorb calcium very readily and once inside the cell the calcium ions are neutralized by membrane phosphates present on the cytosolic side (Melcrová et al., 2016). The binding of calcium ions to the membrane also cause changes in the membrane permeability (Li et al., 2004).

Treatment with divalents or trivalents on ice is followed by treatment with elevated temperature as a heat-shock, which produces a temperature imbalance. Molecules with increased Brownian motion outside the cell are likely to push the DNA molecule inside the cell. However, it is unclear if this kinetic force is sufficient enough to push the adsorbed DNA molecules inside. Panja et al. (2008a) studied the efficacy of cooling and heating cycles by increasing the number of cycles until maximum transformation efficiency was achieved. It was inferred that lowering of temperature actually contributes to protein loss, while heating contributes to lipid loss, and thus together these cycles increase transformation efficiency (Panja et al., 2008a) as it enlarges the pore size on the cell surface. Moreover, due to loss of lipids and proteins, the membrane is depolarized, further reducing the repulsion between the DNA molecule and the membrane. Moreover, cell density can also affect the efficiency of transformation and it has been reported that maximum competency is observed at cell density ranging from 10^7^ to 10^8^ cells ml in the log phase (Taketo, 1974; Norgard et al., 1978).

However, the question remains; whether the pores (through which foreign DNA enters a cell) are formed by the calcium treatment or are they naturally present. There exist natural channels, often called Bayer's bridges, in the membrane that can serve as potential pathway for DNA uptake (Dreiseikelmann, 1994; Sperandeo et al., 2007; Srivastava, 2013). Hanahan (1983) stated that the competent cells have many sites or channels and all these sites and channels have an independent chance of taking part in the uptake of DNA moving toward the process of transformation. All the cells, whether competent or not, compete for the uptake of plasmid but if only competent cells are used for the transformation, the efficiency will be increased up to 50-folds as discussed by Hanahan (1983). The DNA uptake factor is the sum of all the probabilities of DNA uptake through each channel. It was reported that the chances of transformation are not increased by increasing the concentration of DNA but by the increase in the number of channels through which the uptake of DNA takes place (Hanahan, 1983; Nikaido and Vaara, 1985). Moreover, calcium has a dual role in this process; it not only neutralizes the charge but also weakens the cell membrane to produce invaginations (Stein, 1990; Thomas and Rice, 2014).

While it was known that the divalent cations help neutralize the charge, the complex ions can also serve to produce static force of attraction within the DNA molecule. This leads to the folding of DNA into a compact ball-like structure that facilitates its entry into the cell (Clark et al., 2002). A supercoiled ball like structure of the plasmid will have more chances of entering the competent cell for transformation than the extended open circular form of the plasmid. However, if the size of the DNA approaches the size of the pore, the probability of the transformation decreases sharply. When using spermidine or other trivalents, the size of the ball-like structure of DNA might exceed the size of pores in the cell membrane, which can be only solved by altering the physical parameters used in the protocol, primarily the heating and cooling cycles. Whether using divalents or trivalents, their concentrations need to be optimized such that all the phosphates of the DNA are not rendered inaccessible, because some parts of the DNA have to adsorb onto the cell surface and for that free phosphates are required, as inferred by Panja et al. (2008b).

The transformation efficiency is greatly affected by the type of the host cell, as they have different cell surface structures, especially in relation to O-polysaccharides that protrude from the surface of the cell. These surface structures interact with the divalent cations and the DNA, thus making the cell competent for transformation. Different strains of *E. coli*, as discussed above, have been reported to show variance in transformation efficiency, owing to the differences in chemical properties of their cellular envelope (Taketo, 1972). A very dense O-polysaccharide will become a deterrent for the DNA to pass through. However, it has also been claimed that extensive removal of LPS by excessive ethanol-pretreatment reduces transformation efficiency (Roychoudhury et al., 2009). This can be explained by the aforementioned hypothesis that the DNA first attaches to some external component of the cell membrane, which then assists its movement inside the cell. Along with the density, the composition of the O-polysaccahride also plays a role in the reception of the incoming DNA molecule (Lacks, 1977). Moreover, calcium ions also interacts with the membrane and at 100 mM CaCl_2_ concentration, almost all calcium is absorbed by the cell membrane's phosphatidylcholine and phosphatidylserine (Melcrová et al., 2016). Therefore, the membrane properties play a major role in DNA adsorption.

The evidences clearly indicate that the physical and chemical treatments used during transformation, i.e., the temperature imbalances and CaCl_2_ treatment, help deal with the barriers to DNA uptake, such as charge repulsions and pore sizes (Figure 1). Magnesium and calcium combinations are seldom used in transformation protocols, the importance of which should be considered. A combination of divalent and trivalent cations with prolonged incubation times can be suggested to improve the transformation efficiency; as in addition to the charge stabilization, trivalent cations can compact DNA, further aiding its internalization. Bacterial cells could also be grown in presence of CaCl_2_ and MgCl_2_ before inducing competency. Heating and cooling cycles used just once in transformation protocols could also be increased to three times for higher transformation efficiencies. These conditions need to be adjusted and optimized for different bacterial species and strains, owing to the differences in their surface properties. However, there is a need for concrete evidences based on experiments designed exclusively to elaborate this phenomenon.

**Figure 1:**
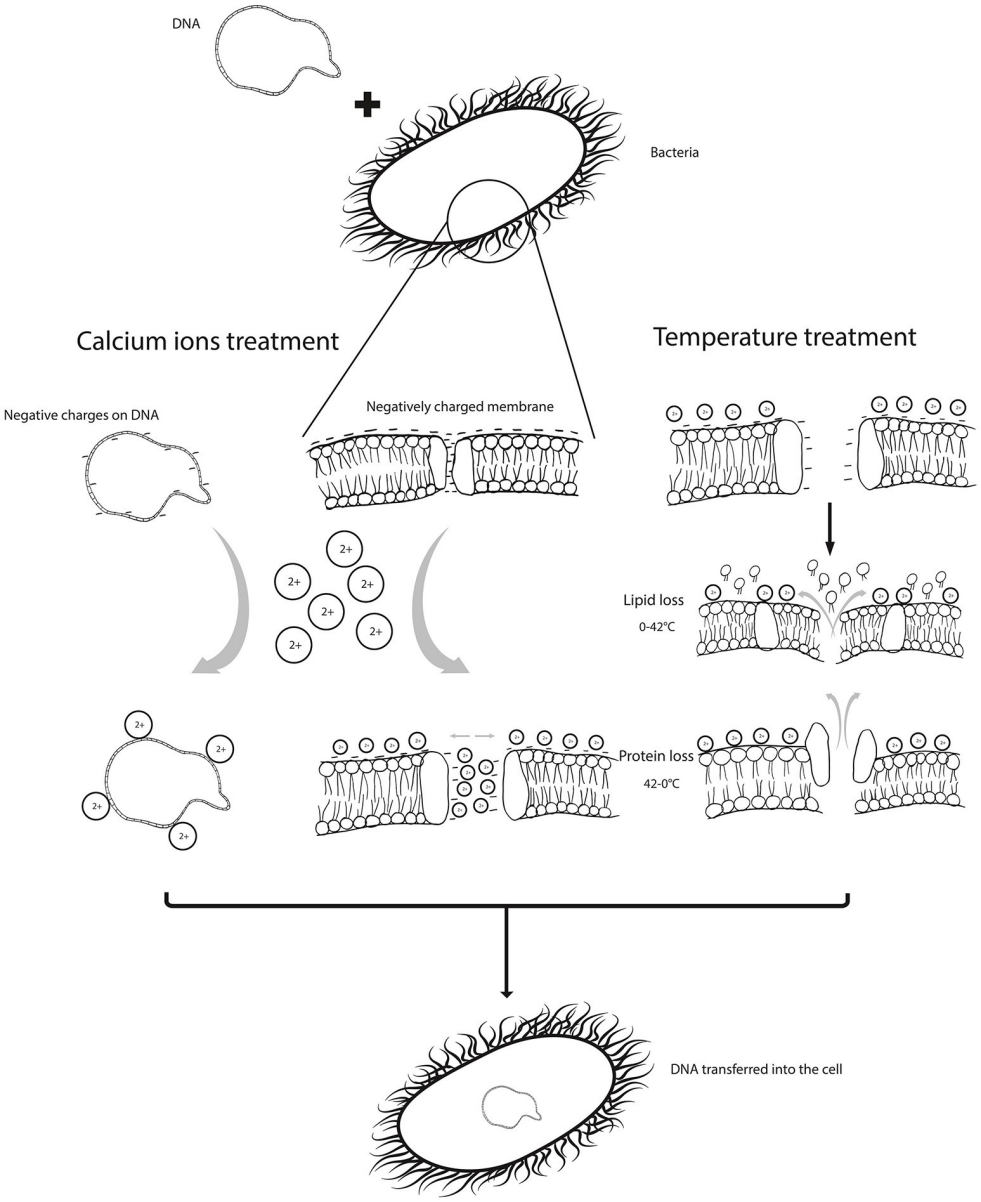
shows the barriers /limitations in uptake of DNA by a bacterial cell, which are; the repulsion caused by negative charges on the cell membrane and DNA and the porosity of the membrane. These are manipulated by chemical treatment, such as calcium ions which neutralize negative charges. Physical parameters can be applied to improve porosity and permeability.

## Author contributions

AA and HM drafted the manuscript. YR put forward the idea of the manuscript and edited the manuscript to the final form. RT helped in the write up of the manuscript.

### Conflict of interest statement

The authors declare that the research was conducted in the absence of any commercial or financial relationships that could be construed as a potential conflict of interest.
